# Why males scar more: hormonal and chromosomal clues to idiopathic pulmonary fibrosis

**DOI:** 10.1186/s13293-026-00942-5

**Published:** 2026-06-25

**Authors:** Sharon J. Elliot, Kristina Clark, Gina Civettini, Ben Roos, Xiaomei Xia, Eva Galdikaite, Simone Pereira-Simon, Austin Kaboff, Paola Catanuto, Sam Grimaldo, Shahriar Shahzeidi, Arthur P. Arnold, Marilyn K. Glassberg

**Affiliations:** 1 Department of Medicine, Loyola University Chicago Stritch School of Medicine, Chicago, IL USA; 2 Stritch School of Medicine, Loyola University Chicago, Chicago, IL USA; 3 Department of Medicine, University of Arizona College of Medicine/Banner, Phoenix, AZ USA; 4 DeWitt Daughtry Family Department of Surgery, University of Miami Leonard M. Miller School of Medicine, Miami, FL USA; 5 Grand Health Institute, Miami, FL USA; 6 Department of Integrative Biology & Physiology, University of California, Los Angeles, CA 90095 USA

**Keywords:** Idiopathic pulmonary fibrosis, Sex differences, Sex chromosomes, Gonadal sex hormones, Four core genotypes, Estrogen receptor, Aging

## Abstract

**Background:**

There is a gap in understanding the predominance of males with idiopathic pulmonary fibrosis (IPF). While gonadal hormones contribute to fibrosis susceptibility, evidence suggests a role for sex chromosomes.

**Methods:**

We used The Four Core Genotypes (FCG) mouse model, which uncouples gonadal sex from sex chromosomes, in aged mice before and after bleomycin (BLM)-induced lung injury. Fibrosis severity was assessed by histology, collagen content, and profibrotic gene expression, along with analysis of estrogen receptor (ER)α and ERβ signaling, matrix metalloproteinase activity, insulin-like growth factor-1 (IGF-1), and microRNAs. Data were analyzed using two-way ANOVA to test effects of gonadal sex, sex chromosome complement, and their interaction; gonadectomy experiments used three-way ANOVA including gonadal status.

**Results:**

BLM-induced lung injury resulted in the greatest fibrosis in XY mice with ovaries, which was associated with elevated ERα expression and increased ERα:ERβ ratio. In contrast, ERβ expression was highest in XX mice with testes and associated with attenuated fibrosis. Multiple fibrotic pathways were regulated by gonadal sex, sex chromosome complement, or their interaction. Gonadectomy revealed organizational and activational effects of sex hormones and uncovers interactions between gonadal sex, sex chromosomes, and hormone status. Sex chromosome–dependent regulation of let-7d and miR-29a linked chromosomal dosage to ERα–IGF-1 mediated remodeling.

**Conclusions:**

These findings identify hormonal and chromosomal mechanisms contributing to sex bias in pulmonary fibrosis and suggest sex-informed therapeutic targets for IPF.

**Graphical Abstract:**

**Figure:**
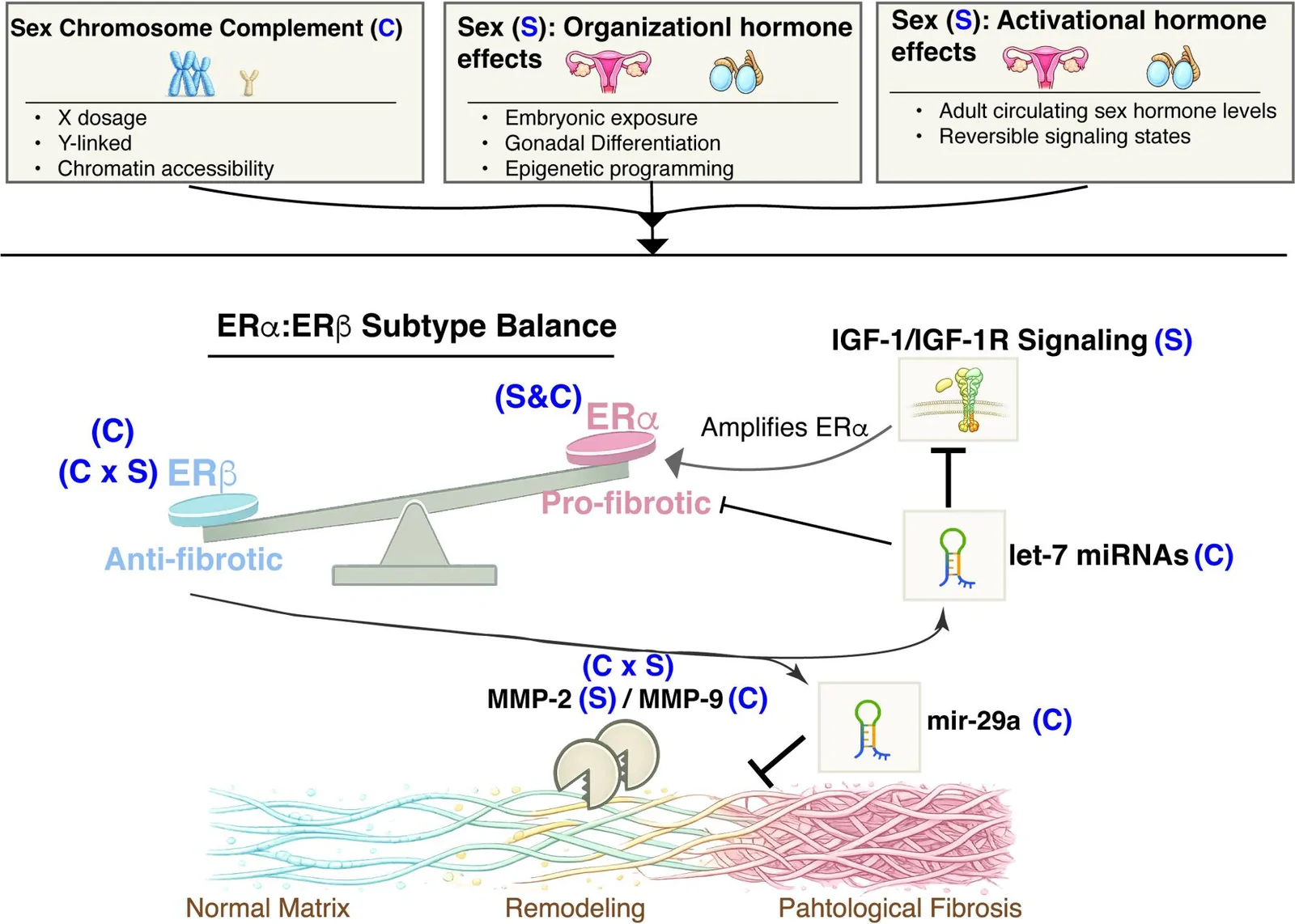

## Abstract (plain language summary)

IPF is a progressive, incurable lung disease characterized by excessive lung scarring and a strong male predominance. The biological reasons for these sex differences are not well understood. Most studies compare males and females as whole groups, which makes it difficult to determine whether these differences are driven by sex hormones, sex chromosomes, or both.

In this study, we used a specialized mouse model that allows us to separate the effects of sex chromosomes (XX or XY) from the effects of male or female hormones. This approach made it possible to test how each factor contributes to lung injury.

We found that both sex chromosomes and sex hormones influence lung injury. Mice with XY chromosomes developed more severe lung injury under certain conditions, even when they did not have male hormones. Removing sex hormones changed the severity of lung injury in ways that depended on the animal’s chromosome makeup. We also identified changes in key molecular pathways that help explain how these biological differences affect lung injury and repair.

Together, these findings show that sex differences in pulmonary fibrosis are not explained by hormones alone. Instead, sex chromosomes and hormones work together to shape disease risk and severity. Understanding these mechanisms may help guide future research toward preventing or treating lung fibrosis in men and women.

## Highlights

Sex differences in pulmonary fibrosis reflect combined effects of sex chromosomes and gonadal sex hormones.

XY chromosome complement increases susceptibility to lung scarring independent of male gonadal hormones.

Gonadectomy reveals both long-lasting organizational and activational effects of gonadal sex hormones and uncovers three-way interactions among gonadal sex, sex chromosomes, and hormone status.

Dissecting chromosomal and hormonal effects provides new insight into the male predominance in IPF.

## Introduction

The prevalence and progression of many chronic diseases differ in females and males [1]. This is true for chronic respiratory diseases including cystic fibrosis [2], COPD [3], asthma [4] and idiopathic pulmonary fibrosis (IPF) [5]. IPF, the most severe and irreversible form of fibrotic lung disease, is diagnosed annually in approximately 50,000 adults aged 60 and older [6, 7]. Risk factors such as male sex, a history of smoking, and advanced age are associated with both increased incidence and reduced survival in IPF.

We previously demonstrated that dysregulated expression of sex hormone receptors was present in lung tissue and myofibroblasts from male subjects with IPF, as well as in the bleomycin (BLM) mouse model of lung injury [8]. However, there are few direct studies to understand the role of sex chromosome complement on the development and progression of IPF [9, 10], although a recent investigation established that mosaic loss of chromosome Y (mLOY) in leukocytes is associated with male susceptibility to IPF [11]. Therefore, we utilized The Four Core Genotypes (FCG) mouse model that produces XX and XY gonadal males (XXM, XYM), and XX and XY gonadal females (XXF, XYF) [12]. In the present study, we assessed fibrosis in aged mice before and after BLM-induced lung injury. We measured changes in body weight, fibrotic markers, insulin-like growth factor (IGF)-1, estrogen receptor (ER)α and ERβ protein expression and microRNAs (miRNA) previously implicated in development of IPF. Since activation of ER subtypes has been shown to regulate matrix metalloproteinases (MMPs)/extracellular matrix accumulation, we also evaluated MMP-9 and MMP-2 activity and miR-29a, an extracellular matrix regulator [13]. We further analyzed activational effects of sex hormones by gonadectomy (GDX) and subsequently reassessed fibrotic pathways.

Our data revealed that some fibrosis-inducing pathways are regulated by an interaction between sex hormones and sex chromosome complement while other aspects are driven by either gonadal hormones or sex chromosome complement alone. These findings underscore the complexity of studying sex differences in chronic disease, as both hormonal and genetic sex components contribute in distinct and overlapping ways.

## Results

### Body weight and fibrotic markers

**Fig. 1:**
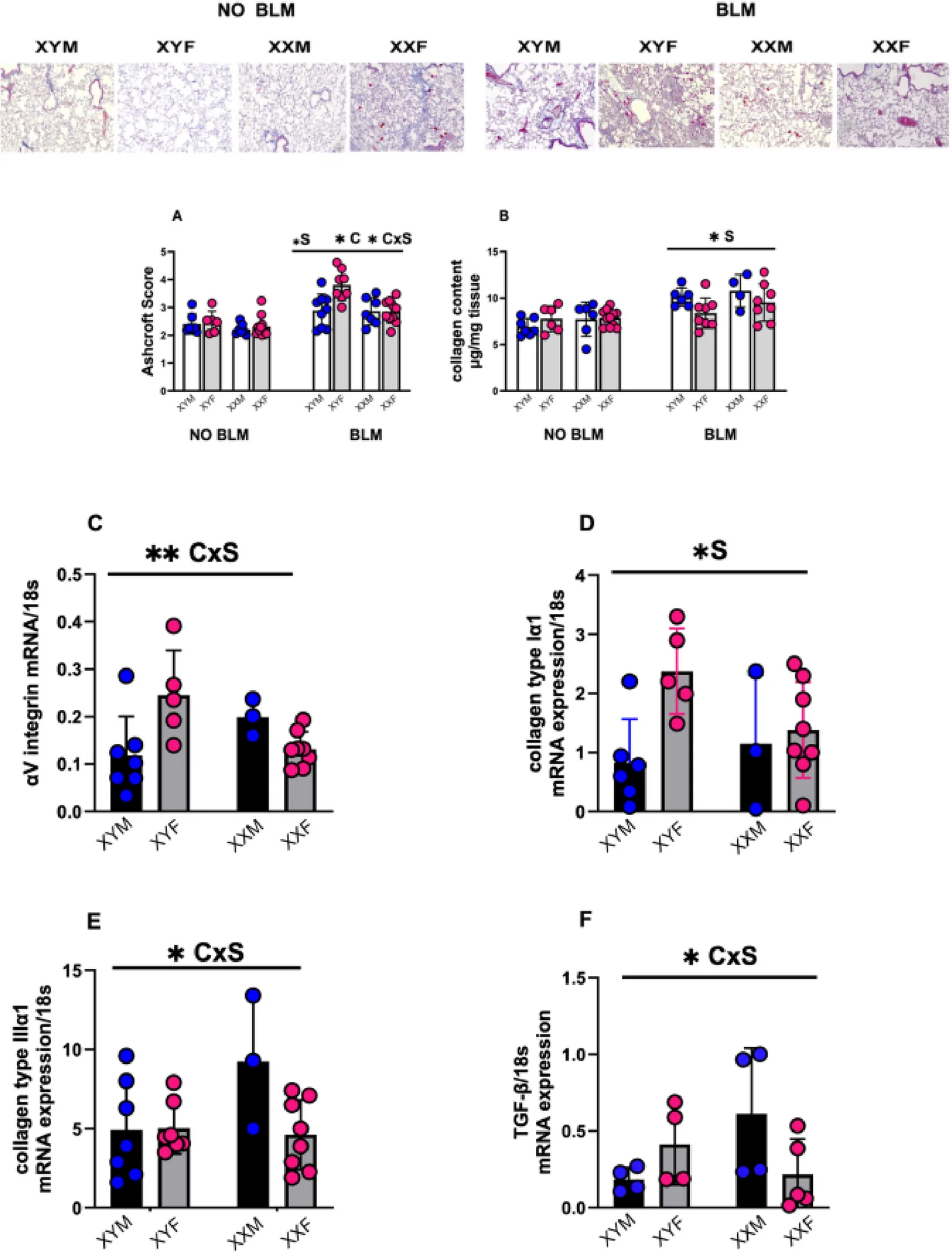
Bleomycin (BLM)-induces increased fibrotic lung injury in XY mice possessing ovarian hormones (XYF). FCG mice were sacrificed at 17 months of age, and pulmonary fibrosis was assessed by semiquantitative Ashcroft score (**A**) and collagen content (**B**) in mice without and with BLM treatment (at sacrifice 21 days post-BLM). Representative lung sections from untreated mice (left panels) and BLM-treated mice (right panels) were stained with Masson’s trichrome (10x; scale bar, 100 μm). Data are graphed as individual biological replicates (*n* = 6–11 mice/group). Pink, gonadal female; blue, gonadal male. Two-way ANOVA was used to assess effects of gonadal sex (S), sex chromosome complement (C), and their interactions as C x S. *p < 0.05, **p < 0.01. BLM induced fibrotic marker mRNA expression is regulated by an interaction between sex chromosome complement (C, XX vs. XY) and gonadal sex (S, gonadal female vs. male) or by gonadal sex alone. Expression of αV integrin (**C**), collagen type I α1 (**D**), collagen type III α1 (**E**) and TGFβ (**F**) mRNA was assessed by qPCR. Data are graphed as individual biological replicates (*n* = 4–8 mice/group). Pink, gonadal female; blue, gonadal male. Results of the two-way ANOVA for gonad-intact mice indicate significant effects (when present) of S (gonadal sex, female vs. male), or C (sex chromosome complement, XX vs. XY). Significant interactions are shown as C x S. *p < 0.05, ** *p* < 0.01

At 16 months of age, the FCG mice cohort was divided into two groups; mice that were administered BLM treatment and those that did not. At baseline, there were no group differences in body weight (BW). BLM-induced lung injury was confirmed by BW loss at sacrifice across all groups (Table 1) with the greatest BW loss in gonadal females (XYF and XXF) compared with gonadal males (S **p < 0.01). Lung sections were stained with Masson’s trichrome, and pulmonary fibrosis was measured by semiquantitative Ashcroft scale. There was no evidence of significant fibrosis or difference in collagen content between groups in mice that did not receive BLM treatment (Fig. 1A and B). BLM administration increased Ashcroft scores in all groups compared to untreated mice (BLM effect ***p < 0.001). BLM-induced a difference in Ashcroft scores among groups due to a significant C x S interaction (*p < 0.05), with additional main effects of S and C (*p < 0.05, Fig. 1A). At day 21 post-BLM, lungs from XYF mice had a higher Ashcroft score (3.8 ± 0.5, *N* = 8) than XYM (2.8 ± 0.5, *N* = 9), XXF (2.8 ± 0.4, *N* = 10) or XXM (2.8 ± 0.4 *N* = 7, **p < 0.01, Fig. 1A), indicating more severe fibrotic injury, which was consistent with greater weight loss observed in this group. While all lungs exhibited some patchy fibrosis, XYF lungs exhibited interstitial fibrosis with increased macrophages. Notably, XYF mice did not exhibit elevated baseline lung collagen levels (Fig. 1B). Further, BLM treatment exhibited an S effect on collagen content with higher levels in gonadal males than gonadal females (*p < 0.05, Fig. 1B), indicating that post-injury collagen accumulation is more closely associated with gonadal sex rather than sex chromosome complement.

**Table 1:** Body weight and BLM-induced weight loss in age FCG mice

	XYM *N*=18	XYF *N*=17	XXM *N*=13	XXF *N*=15
Body Weight (grams) intact mice (17 months) no BLM	27.8±3.7	26.6±7.1	28.0±2.6	31.8±5.0

	XYM *N*=10	XYF *N*=9	XXM *N*=5	XXF *N*=11
Body weight loss (grams) post BLM- induced injury	4.2±3.7	7.0±3.5	2.7±0.7	6.3±2.3

At the transcriptional level, α_V_-integrin expression increased in a manner dependent on the interaction between sex chromosome complement and gonadal sex (C x S **p < 0.01, Fig. 1C). Col1α1 mRNA expression differed primarily by gonadal sex (S *p < 0.05, Fig. 1D), with higher expression observed in XYF mice. Col3a1 expression was increased in XXM, whereas XXF and all XY mice exhibited similar expression levels, consistent with a C x S interaction (*p < 0.05, Fig. 1E). TGFβ mRNA expression was higher in XYF mice compared to XYM (*p < 0.01, Fig. 1F), and exhibited a pattern consistent with a C x S interaction. Expression of all measured transcripts increased across all groups of mice (Table 2), consistent with prior reports in aging C57BL6 mice [14–18]. The groups that did not receive BLM represent baseline expression levels, while BLM treatment induces lung injury. Therefore, subsequent analyses focus on BLM treated animals to evaluate genotype-dependent differences in the response to injury. Together, Fig. 1 reveals that distinct components of the fibrotic response post BLM are differentially associated with gonadal sex and sex chromosome complement. While fibrosis severity and select signaling markers exhibited C x S interactions, collagen content and Col1α1 mRNA expression were primarily linked to gonadal sex, and Col3a1 expression displayed a unique interaction pattern in XXM mice.

**Table 2:** Lung tissue mRNA expression/18s ratio without BLM and post-BLM

No BLM	XYM	XYF	XXM	XXF
α_V_-integrin	0.02±0.02 *N*=8	0.04±0.04 *N*=6	0.03±0.03 *N*=6	0.02±0.03 *N*=12
Collagen type Iα1	0.18±0.2 *N*=6	0.13±0.06 *N*=7	0.25±0.19 *N*=5	0.08±0.06 *N*=12
Collagen type3α1	0.8±0.09 *N*=6	0.33±0.18 *N*=4	0.08±0.06 *N*=3	0.13±0.10 *N*=8
TGFβ	1.5±1.6 *N*=8	2.1±1.9 *N*=6	2.3±2.8 *N*=6	1.2±1.1 *N*=12

BLM	XYM	XYF	XXM	XXF
α_V_-integrin	0.11±0.08 *N*=7	0.2±0.09 *N*=5	0.19±0.03 *N*=3	0.13±0.03 *N*=8
Collagen type Iα1	0.8±0.7 *N*=6	2.3±0.7 *N*=5	1.1±1.1 *N*=3	1.3±0.8 *N*=8
Collagen type3α1	4.9±3.0 *N*=7	5.0±1.6 *N*=7	9.2±4.2 *N*=3	4.6±2.2 *N*=8
TGFβ	14.4±5.1 *N*=8	28.2±8.2 *N*=8	18.6±12.7 *N*=3	13.4±7.8 *N*=9

### Estrogen receptor expression

**Fig. 2:**
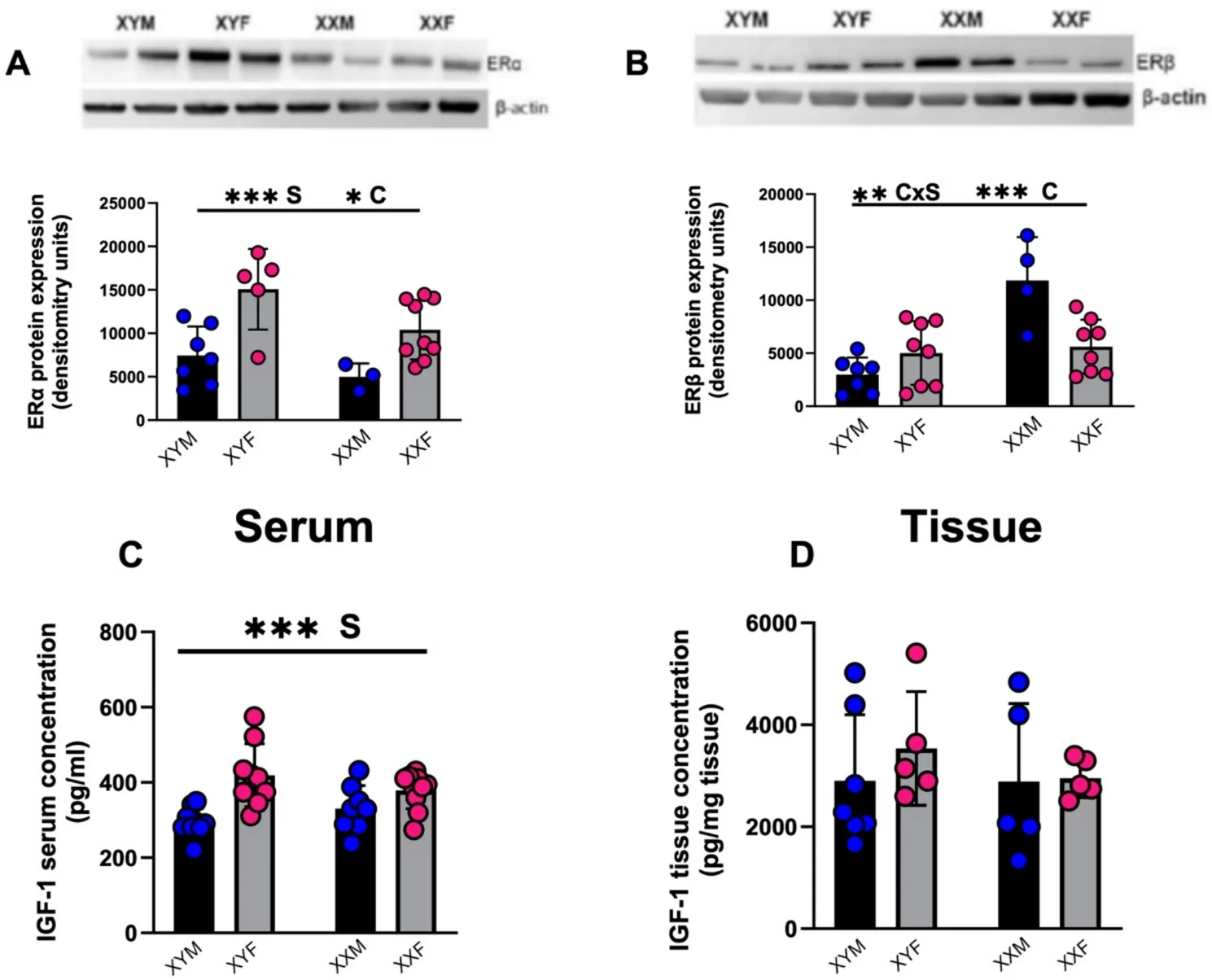
Protein expression of estrogen receptor (ER)α and ERβ and serum and tissue expression of insulin like growth factor (IGF)-1. Western blot analysis of ERα (**A**) and ERβ (**B**) protein expression was performed on lung tissue collected 21 days following BLM-induced lung injury. Insets show representative Western blots. Data are graphed as individual biological replicates (*n* = 3–9 mice/group). Pink, gonadal female; blue, gonadal male. Two-way ANOVA assessed effects of gonadal sex (S), sex chromosome complement (C), and their interaction (C x S). *p < 0.05, ** *p* < 0.01, ***p < 0.001. IGF-1 expression is highest in the serum of XYF mice and is regulated by gonadal sex. IGF-1 was measured by ELISA in serum (**C**) and lung tissue (**D**) of FCG mice. No differences were observed in lung tissue IGF-1 expression between groups. Data are graphed as individual biological replicates (*n* = 5–10 mice/group). Pink, gonadal female; blue, gonadal male. Two-way ANOVA assessed effects of gonadal sex (S). ***p < 0.001

Before BLM administration, lung tissue ERα expression differed by gonadal sex and sex chromosome complement with the highest levels observed in XYF mice. However, ERβ expression did not differ between groups (data not shown). In mice that received BLM, lung tissue ERα protein expression was highest in XYF compared to XYM and XXM groups (S ***p < 0.001, C*p < 0.05, Fig. 2A). This was associated with a higher ERα:ERβ ratio and greater fibrosis severity in XYF mice. In contrast, ERβ protein expression was highest in lung tissue from XXM mice (Fig. 2B). Two-way ANOVA revealed an effect of C (***p < 0.001) and a C x S interaction (**p < 0.01, Fig. 2B).

### IGF-1 expression

IGF-1 expression was measured in serum and lung tissue, based on prior evidence linking IGF-1 to fibrotic responses [8]. Serum IGF-1 levels were higher in gonadal females compared to gonadal males, with the highest levels observed in XYF mice (S, ***p < 0.001, Fig. 2C). In contrast, no differences were observed between groups in lung tissue IGF-1 expression (Fig. 2D).

### Matrix metalloproteinases (MMPs)

**Fig. 3:**
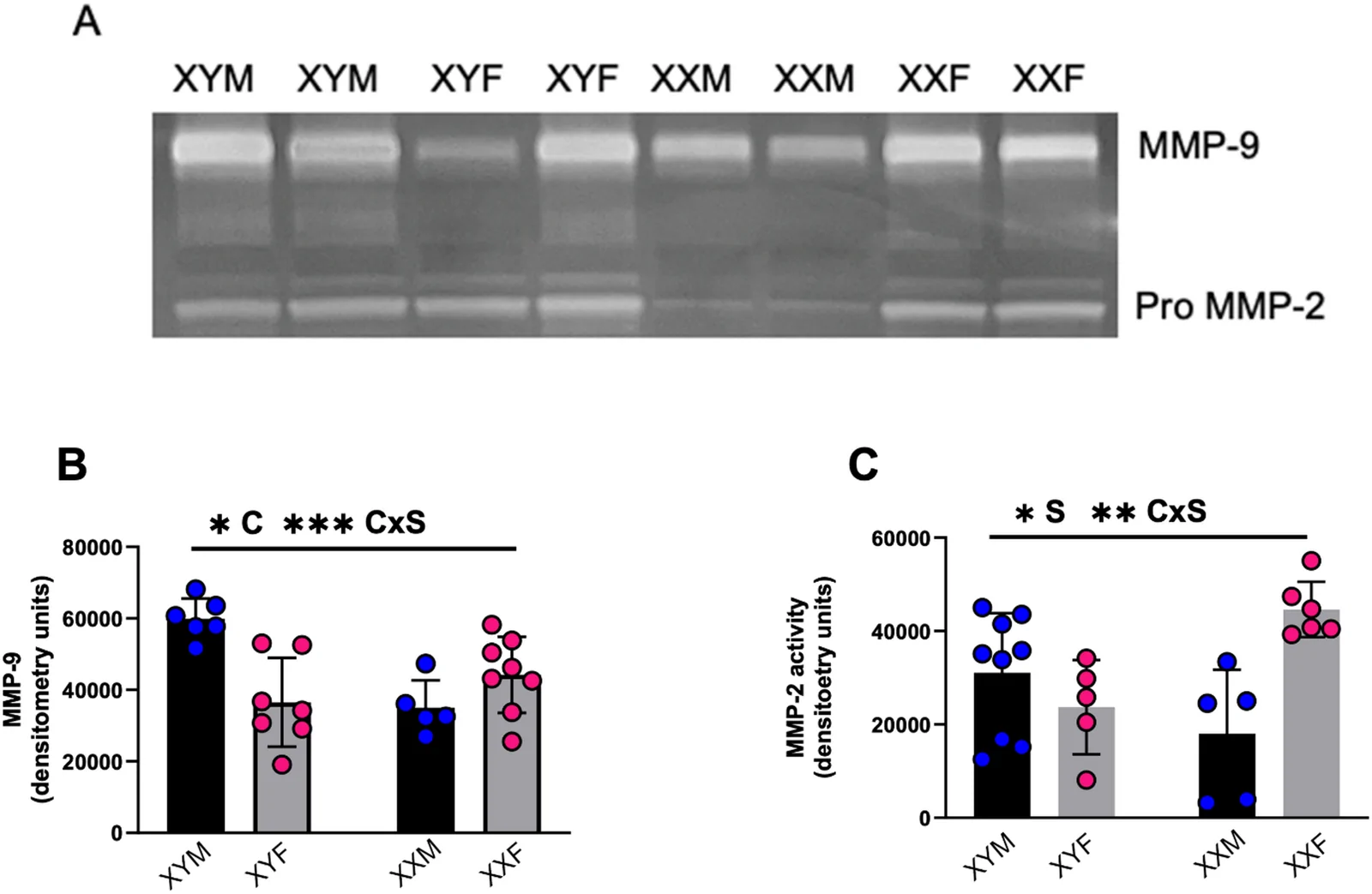
Matrix metalloproteinases (MMP)-2 and MMP-9 activity following BLM-induced lung injury is differentially regulated by gonadal sex and sex chromosome complement. MMP-9 (**A**) and MMP-2 (**B**) activity were determined by zymography. Insets show representative zymograms. Data are graphed as individual biological replicates (*n* = 5–9 mice/group). Pink, gonadal female; blue, gonadal male. Two-way ANOVA assessed effects of gonadal sex (S), sex chromosome complement (**C**), and their interactions (C x S). *p < 0.05, **p < 0.01, ***p < 0.001

MMP-9 activity was highest in XYM mice and was associated with a main effect of C (*p < 0.05) and C × S interaction (**p < 0.001, Fig. 3A). In contrast, MMP-2 activity was highest in XXF mice and was associated with a main effect of S (*p* < 0.05) along with a C × S interaction (**p < 0.01; Fig. 3B).

### MicroRNA expression

**Fig. 4:**
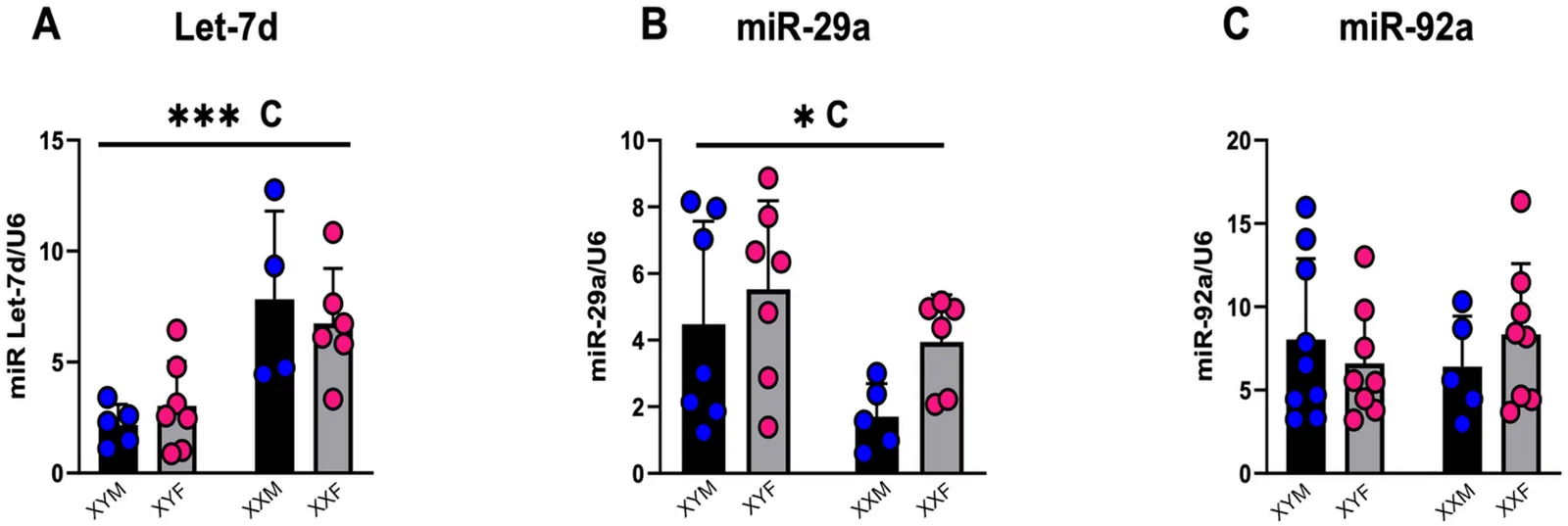
Lung tissue miRNA expression of let-7d and miR-29a following BLM-induced lung injury is regulated by sex chromosome complement, whereas miR-92 is not. Expression of let-7d (**A**), miR-29a-5p (**B**), and miR-92a-5p (**C**) was determined by qPCR. Data are graphed as individual biological replicates (*n* = 4–9 mice/group). Pink, gonadal female; blue, gonadal male. Two-way ANOVA assessed effects of sex chromosome complement (C). *p < 0.05, ***p < 0.001

We next analyzed a selected panel of microRNAs previously implicated in fibrotic lung disease [19]. Following BLM-induced injury, let-7d (XX > XY) and miR-29a (XY > XX) exhibited sex chromosome-dependent regulation, but in opposite directions (*p < 0.05, ***p < 0.001, Fig. 4A and B). However, miR-92a expression did not differ between groups (Fig. 4C).

### Effects of gonadectomy

GDX was performed at 16 months and mice were allowed to recover prior to BLM-induced lung injury. Circulating testosterone levels decreased after GDX (Table 3). Serum 17β-estradiol, was below the ELISA detection limits (5-3200pg/ml) in all groups, consistent with aged female mice in reproductive senescence [20] and low circulating estradiol in males [21]. Our goal was to determine whether the effects of gonadal sex or sex chromosome complement depend on circulating gonadal hormone levels at 16 months. GDX reduced the effect of BLM-induced BW decrease in XYF with lesser effect in the other groups (Table 3). In intact mice, serum IGF-1 levels were higher in gonadal females. However, following GDX, the gonadal sex effect was diminished, and a significant interaction between sex chromosome complement and gonadal sex emerged (C x S **p < 0.01, Table 4).

**Table 3:** Serum testosterone levels (ng/ml) in intact and gonadectomized FCG mice

Genotype	XYM *N*=6	XYF *N*=7	XXM *N*=8	XXF *N*=6
Intact	0.37±0.12	0.23±0.07	0.41±0.24	0.22±0.79

Genotype	XYM *N*=3	XYF *N*=3	XXM *N*=4	XXF *N*=3
GDX	0.18±0.06	0.15±0.05	0.26±0.12	0.16±0.05

**Fig. 5:**
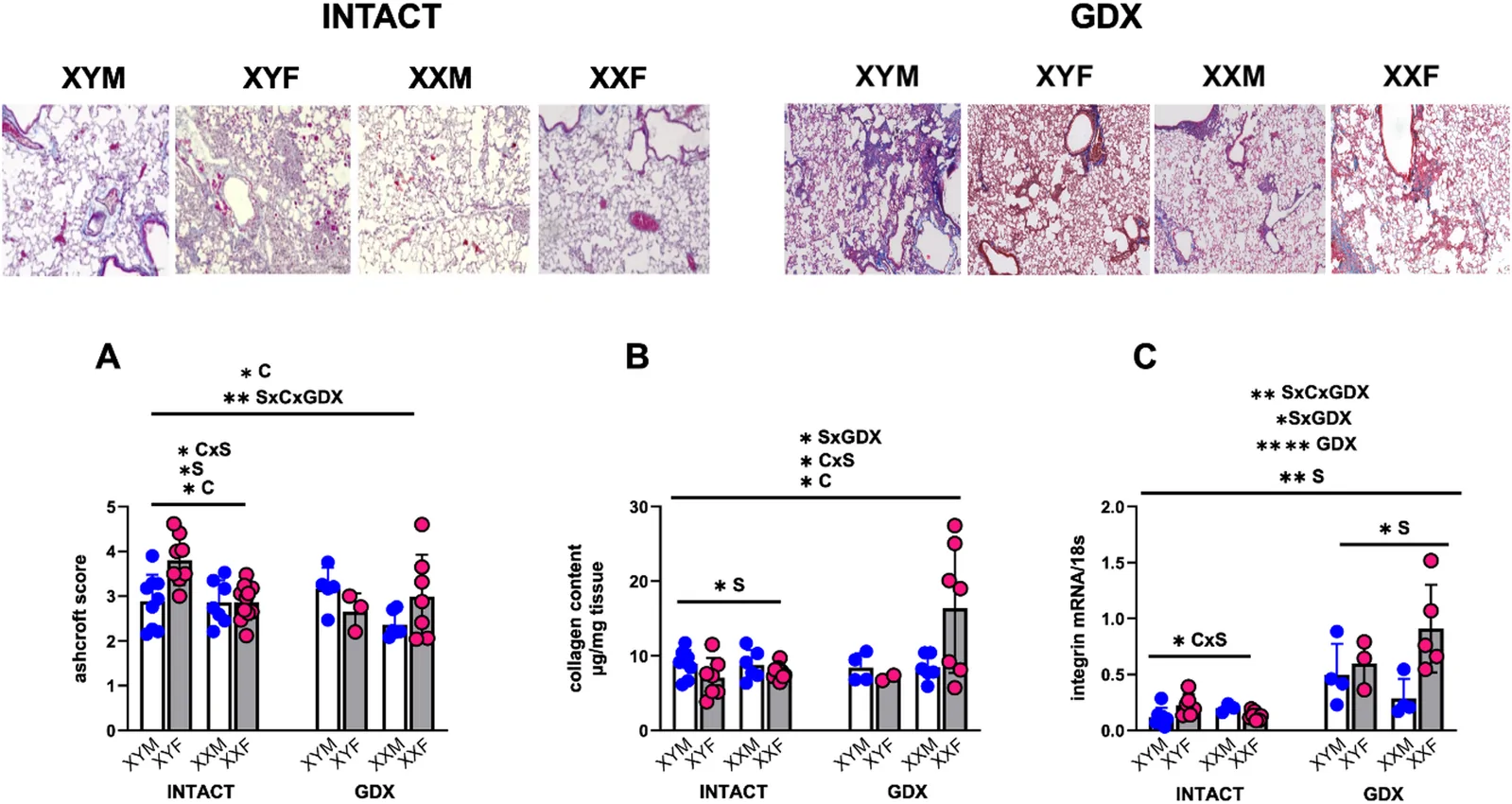
Gonadectomy (GDX) induces interactions that modulate fibrotic marker expression across all groups. Ashcroft score (**A**), collagen content (**B**), and integrin mRNA expression (**C**) were determined at sacrifice 21 days following BLM-induced injury in intact and GDX FCG mice. Representative histological lung sections from intact (left) and GDX mice (right) were stained with Masson’s trichrome (10x; scale bar, 100 μm). Data are graphed as individual biological replicates (*n* = 3–11 mice/group). Pink, gonadal female; blue, gonadal male. Three-way ANOVA assessed effects of gonadal status (G; intact vs. GDX), gonadal sex (S), sex chromosome complement (**C**), and their interactions. Two-way ANOVAs were also performed for gonad-intact mice and GDX separately. Horizontal lines indicate groups included in each statistical comparison. *p < 0.05, **p < 0.01, ****p < 0.0001

Analysis of Ashcroft score, collagen content, and α_V_ integrin mRNA expression revealed three-way interactions, indicating that presence or absence of gonads (intact vs. GDX) modified the association between sex chromosome complement and gonadal sex in fibrotic severity and related signaling (G x C x S, **p < 0.01, Fig. 5A, C and G x S, *p < 0.05, Fig. 5B).

**Table 4:** Effects of gonadectomy on BW loss (grams) and serum IGF-1 post BLM-induced lung injury

Genotype	XYM *N*=10	XYF *N*=9	XXM *N*=5	XXF *N*=11
Intact BW loss	4.2±3.7	7.0±3.5	2.7±0.7	6.3±2.3

Genotype	XYM *N*=4	XYF *N*=3	XXM *N*=4	XXF *N*=8
GDX BW loss	5.8±3.9	1.6±1.4	3.4±1.7	6.2±4.5
Serum IGF-1 (ng/ml)				

Genotype	XYM *N*=9	XYF *N*=9	XXM *N*=8	XXF *N*=10
Intact	293.9±37.9* ###	419.1±83.7&,@	330.3±61.4	378.7±48.5
&p<0.05 XYF vs XXM, *p<0.05*XYM vs XXF, ###p<0.001 XYM vs XYF				

Genotype	XYM N=4	XYF N=3	XXM N=4	XXF N=4
GDX	303.2±31.6	288.6±49.1	357.5±43.8	352.3±65.0
@p<0.05 XYF intact vs XYF GDX and XYM GDX				

**Fig. 6:**
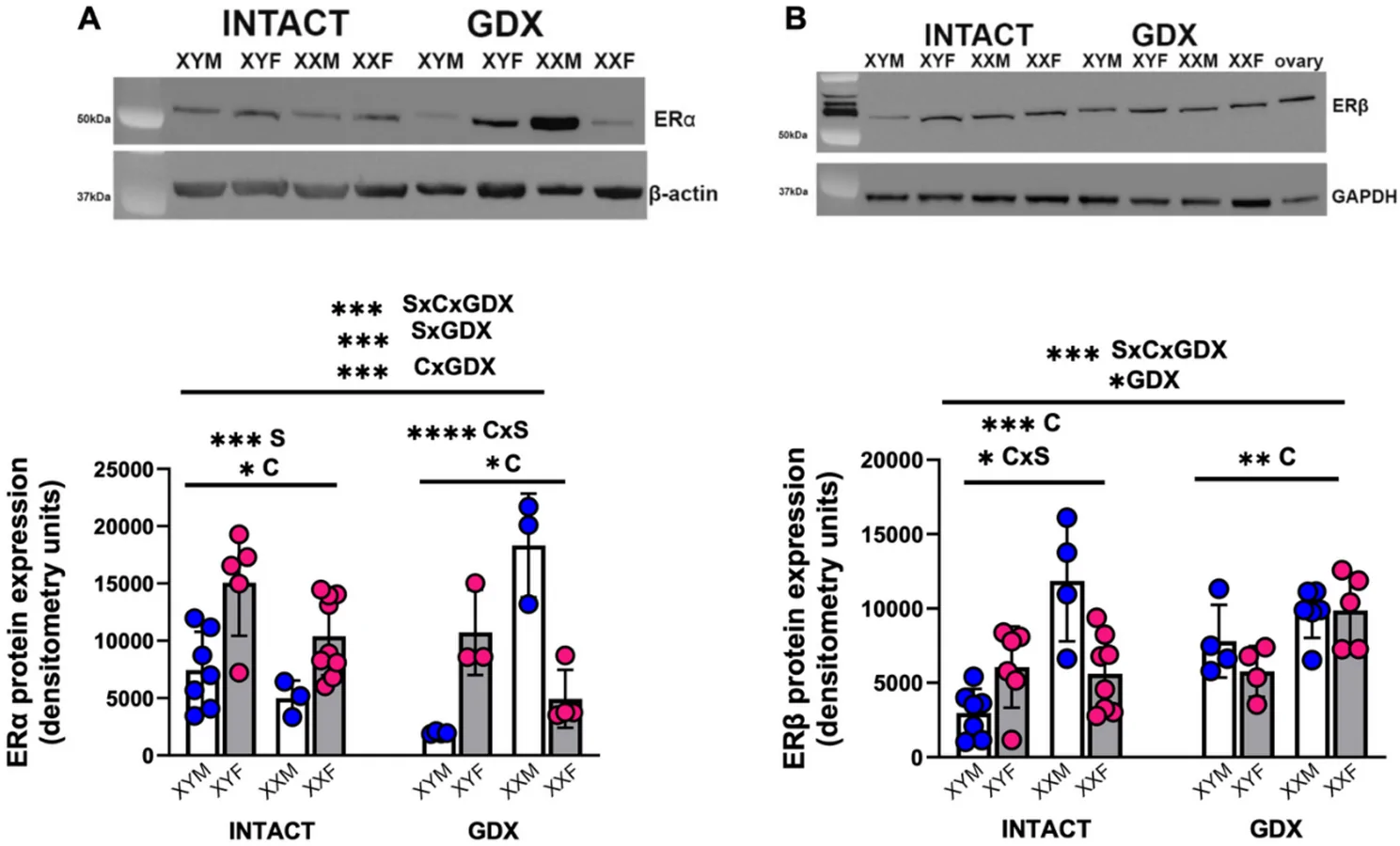
Lung tissue estrogen receptor (ER) subtype expression is regulated by gonadectomy (GDX). Western blot analysis of ERα (**A**) and ERβ (**B**) protein expression was performed on lung tissue. Insets show representative Western blots. Data are graphed as individual biological replicates (*n* = 3–9 mice/group). Pink, gonadal female; blue, gonadal male. Three-way ANOVA assessed effects of gonadal status (G; intact vs. GDX), gonadal sex (S), sex chromosome complement (C), and their interactions. Two-way ANOVAs were also performed for gonad-intact mice and GDX mice separately. Horizontal lines indicate groups included in each statistical comparison. *p < 0.05, **p < 0.01, ***p < 0.001, ****p < 0.0001

ERα and ERβ expression were altered after GDX in a three-way interaction between gonadal sex, sex chromosome complement, and GDX (C x S x G ***p < 0.001, Fig. 6A and B). In XYF mice, GDX reversed the ERα:ERβ subtype ratio in XYF mice in concert with a lower Ashcroft score suggesting the importance of the contribution of sex chromosome and sex hormone interaction in activation of fibrotic pathways in the aged lung.

**Fig. 7:**
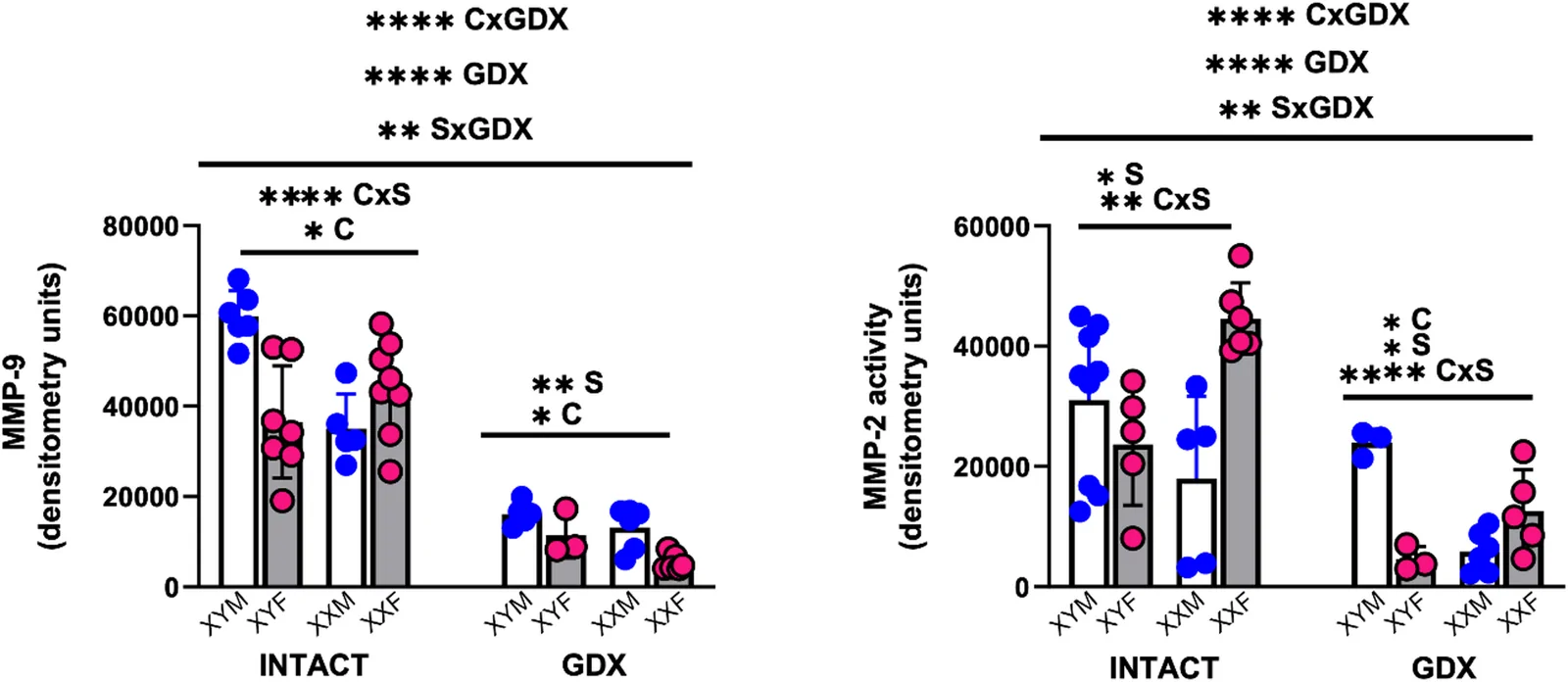
Circulating gonadal sex hormones actively maintain basal matrix metalloproteinase (MMP)-9 and MMP-2 activity in aging mice. MMP activity was determined by zymography. Data are graphed as individual biological replicates (*n* = 3–9 mice/group). Pink, gonadal female; blue, gonadal male. Three-way ANOVA assessed effects of gonadal status (G; intact vs. GDX), gonadal sex (S), sex chromosome complement (C), and their interactions. Two-way ANOVAs were also performed for gonad-intact mice and GDX mice separately. Horizontal lines indicate groups included in each statistical comparison. *p < 0.05, **p < 0.01, ***p < 0.001, ****p < 0.0001

**Fig. 8:**
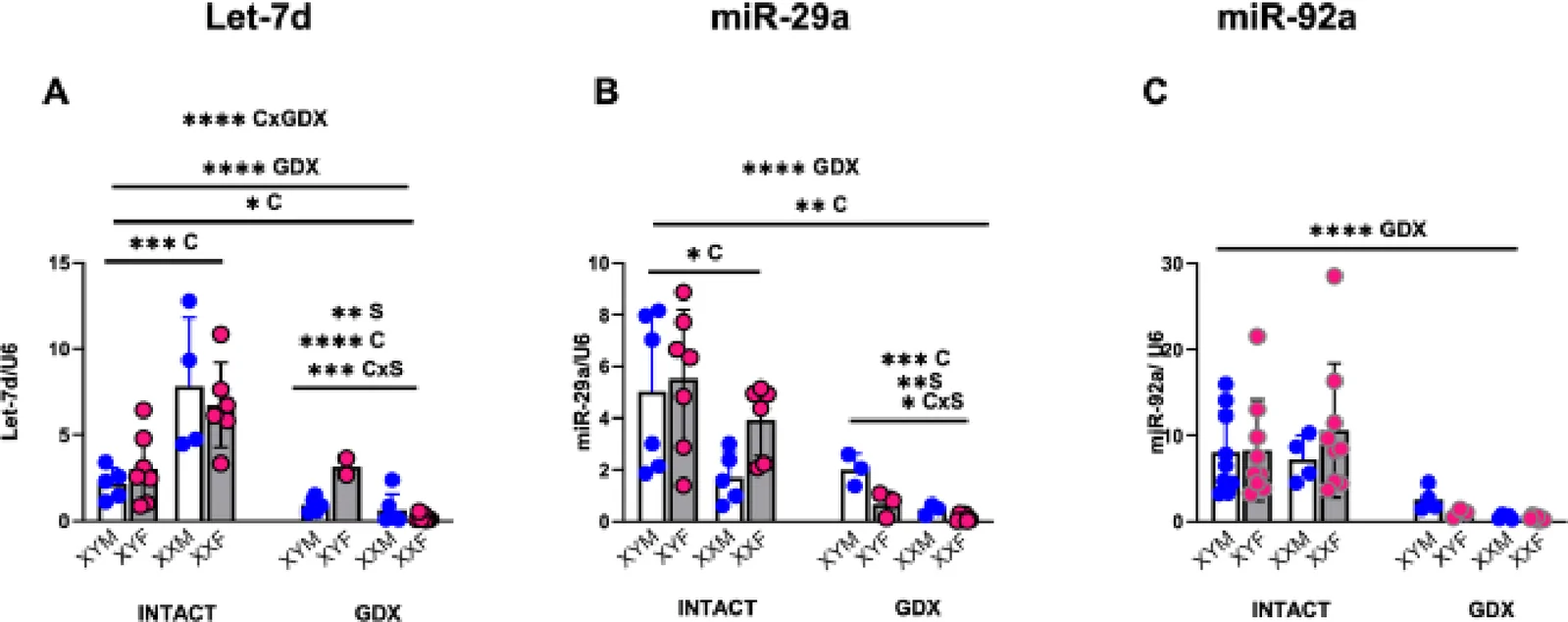
Lung tissue microRNA (miRNA) expression decreases across all groups after gonadectomy (GDX). Data are graphed as individual biological replicates (*n* = 3–12 mice/group). Let-7d (**A**), miR-29a (**B**) and miR-92a (**C**). Pink, gonadal female. Blue, gonadal male Results of the 3-way ANOVA for all groups (horizontal line spanning all groups), and of 2-way ANOVAs for gonad-intact alone or GDX alone (horizontal lines above spanning only each condition), indicate significant effects (when present) of G (gonadal status, intact vs. GDX), S (gonadal sex, female vs. male), or C (sex chromosome complement, XX vs. XY) or interactions. *p < 0.05, **p < 0.01, ***p < 0.001, ****p < 0.0001

MMP-9 activity was altered by gonadectomy in a genotype-dependent manner, with significant interactions between GDX and sex chromosome complement (C x G, ****p < 0.0001) and between GDX and gonadal sex (S x G, **p < 0.01, Fig. 7A). MMP-2 activity also differed between intact and GDX mice, reflecting an interaction between sex chromosome complement and gonadal status (C x G, ****p < 0.0001, Fig. 7B). Given that gonadal status altered enzymatic activity, we next assessed microRNA expression following BLM-induced lung injury. GDX decreased expression of let-7D selectively in XX mice (C x G, ****p < 0.0001, Fig. 8A). In contrast, expression of miR-29a and miR-92a were decreased by GDX across all groups (Fig. 8B and C). 

### Discussion

Although most sex differences have been attributed to distinct effects of gonadal hormones, increasing evidence indicates that sex-biased effects of X or Y genes also contribute [22–26]. Our study demonstrates for the first time that gonadal sex, sex chromosome complement, and the effects of circulating gonadal hormones (which we removed by GDX) influence susceptibility to lung injury in aging FCG mice. These findings highlight the importance of studying the contributions of sex chromosome and gonadal sex to age-associated pulmonary injury.

We found that collagen content and Col1α1 mRNA expression were regulated in a sexually dimorphic manner after BLM-induced lung injury. Other markers including Ashcroft score, αV-integrin, Col3α1, and TGFβ mRNA expression were regulated in a chromosomal manner or the interaction between sex chromosome complement and gonadal hormones. ER subtypes, MMP activity, IGF-1 expression, and profibrotic microRNAs were also regulated either by sex chromosomes, gonadal hormones or interaction of both.

To further unravel these effects, we performed GDX on 16 month-old mice. Since the mice were aged at sacrifice, differences between groups of gonadal females and gonadal males reflect organizational gonadal hormone effects (those occurring at an earlier age prior to gonadectomy), whereas activational (reversible) effects of hormones are seen as phenotypic differences between intact and GDX mice [27]. MiR-92a showed no effects of gonadal hormones or sex chromosomes in intact animals, but decreased uniformly across groups after GDX, indicating that the lack of sex difference was because of balanced effects of ovarian and testicular hormones (Fig. 8). In a comparison with intact mice, all other measured indices of fibrosis and fibrotic inducing pathways were regulated by GDX in a manner that depended on either gonadal type or sex chromosome complement, or both. In some cases (α_V_-integrin mRNA levels, Fig. 5 and MMP-2 activity, Fig. 7), the effects of gonadal hormones were seen even after removal of the gonads, indicating that these effects were likely more long lasting, caused by gonadal hormones before the gonads were removed. These results reveal that sex differences in effects of BLM-induced lung injury are caused by sex chromosome complement and acute and long-lasting effects of gonadal hormones. This is consistent with our and other studies highlighting sex-biasing factors acting separately or together to influence injury and fibrosis in the heart, lung, and pulmonary vasculature [28–31]. Future studies performing gonadectomy earlier in life could help distinguish developmental (“organizational”) effects of sex hormones from later-life (“activational”) hormonal influences on fibrotic susceptibility within the FCG model.

GDX-induced divergent regulation of ER subtype expression. ERα expression increased and ERβ expression decreased in XXM mice (GDX). In contrast, intact XYF mice had the highest ERα expression and the highest fibrosis score, while ERβ expression was elevated in intact XXM mice potentially contributing to protective antifibrotic signaling [32, 33]. Because ERα activation has been shown to regulate profibrotic pathways in IPF [8], our findings suggest that sex chromosome complement may function as a modifier of estrogen-dependent fibrotic susceptibility. These data coupled with high Ashcroft score, increased α_v_ integrin, and Col1 mRNA expression further support the role of ERα pathway signaling as a critical step in fibrosis regulation.

We established that IGF-1 was responsible for ER activation and downstream fibrotic induction (regulation of collagens, MMPs and TGFβ signaling) in myofibroblasts isolated from individuals with IPF and lungs from the aging BLM-induced lung injury model [8]. The increased serum IGF-1 expression in XYF mice coupled with high ERα, suggested that crosstalk between IGF-1 and ERα [34] could enhance ERα ligand-independent fibrotic signaling in the FCG model. The gonadal effect on IGF-1 before GDX and the sex chromosome effect after GDX suggest that age-associated decline in sex steroid hormones and/or signaling may amplify ERα-IGF-1 mediated pathways. In addition, a prior study by Hernandez et al., reported that IPF pathogenesis was dependent on TGFβ-IGF-1 crosstalk [35], which likely is active in the FCG model as well.

The regulation of MMP-9 in a sex chromosome and gonadal hormone-dependent manner underscored the integrated influence of chromosomal sex and gonadal hormone status on ECM turnover. The decrease in both MMP-9 and MMP-2 by removal of gonads suggested that circulating sex steroids actively maintain basal MMP expression even in aging mice. MMP gene transcription can be directly regulated by estrogen stimulation of ERα and androgen stimulation via the androgen receptor [36, 37]. Alternatively, promoter response elements and signaling cascades such as AP-1, NF-κB, and TGF-β/SMAD can also regulate MMPs [38, 39]. MMP-9 and − 2 are upregulated in lung tissue isolated from subjects with IPF, especially in areas of ECM remodeling as well as during infiltration of inflammatory cells [40, 41]. Since MMP expression is controlled at multiple levels, including growth factor signaling and sex steroid hormone signaling, the removal of circulating gonadal hormones after GDX could directly reduce MMP-2/-9 expression, but also disrupt the hormone growth factor crosstalk that represses TGF-β and IGF-1 signaling. This integrated framework provides a potential mechanism for how sex chromosome complement and age-related declines in sex steroids may converge to enhance fibrosis susceptibility in IPF.

Sex chromosome complement regulation of let-7d and miR-29a could occur through dosage effects, chromatin accessibility, or sex-linked transcriptional regulators [42–44]. Let-7a and 7d repress ERα and profibrotic pathways including TGF-β signaling [8] and regulate IGFR by binding to its 3’ UTR [45]. Therefore, decreased let-7d could lead to increased IGF-1R expression and activation by elevated IGF-1. Activation of the IGFR by IGF-1 may induce ECM and fibroblast proliferation directly through AKT and MAPK or through indirect activation of ERα profibrotic pathways. The importance of in vivo fibroblast proliferation was recently demonstrated through lineage tracing in mice and fibrotic human lung tissue [46] suggesting a role for these pathways since individuals with IPF have increased IGF-1 expression [35, 47, 48]. Finally, miR-29a which represses collagen isoforms (col1α1 and col3α1) inhibits matrix deposition [13, 49]. We postulate that miRNAs could function as intermediaries linking sex-chromosomal regulation to pathogenic remodeling in the lung. GDX also lowered miRNAs studied across all groups, indicating that residual hormone levels play a role in their basal expression.

Taken together, these findings underscore the integrated effects of gonadal hormonal signaling and sex chromosome complement on fibrosis susceptibility and may contribute to the male predominance observed in IPF. Our data reveal that sex chromosome complement contributes to susceptibility to lung injury through Y-linked and X-dosage–dependent mechanisms that interact with gonadal hormonal signaling. In addition, the presence of ovaries may exacerbate fibrotic pathways, as reflected by increased fibrosis in XYF mice compared with other groups. Consistent with this, evidence from human studies also supports a role for sex chromosomes in IPF pathogenesis. Mosaic loss of chromosome Y (mLOY) in aging men has recently been associated with an elevated risk of IPF and other age-associated disorders [11, 50, 51]. Mechanistically, mLOY results in the loss of Y-linked chromatin regulatory genes, which may alter transcriptional programs and promote upregulation of profibrotic pathways including TGFβ and IGF-1 signaling [52, 53], further repressing antifibrotic miRNAs such as let-7. In XYF mice, which retain the Y chromosome but have ovaries, low levels of androgens combined with Y-linked factors and/or single-dose X gene effects, and increased ERα activity may promote increased TGFβ and IGF-1 signaling and fibrosis progression [11, 50, 51, 52, 53].

In regard to sex hormones, Mendelian randomization analyses demonstrated that higher genetically predicted testosterone protects against the risk of IPF [54]. In addition, increased estrogen sulfotransferase in aging men may inactivate estrogens. As testosterone decreases with age while estrogen remains stable a reduced T: E ratio may occur [55, 56] and a reduction of antifibrotic influence. This hormonal shift, together with increased IGF-1 signaling, could amplify ERα-mediated mechanisms, even in the presence of less active estrogen. Collectively, these pathways provide a fibrotic environment that may contribute to the development and progression of fibrosis.

In conclusion, this study identifies complex processes because of the interactions of gonadal hormones and sex chromosome complement. Recently, an official ATS research statement provided suggestions to address sex differences in preclinical models of lung disease. The authors proposed investigations of the long-lasting effects of hormones, sex differences in aging and effects of sex chromosomes [57]. Our work provides initial data to guide future mechanistic studies in the understanding of male predominance in age-associated fibrotic lung disease.

## Methods

### Sex as a biological variable

Sex was considered as a biological variable and directly investigated using the FCG model, which dissociates gonadal sex from sex chromosome complement. Male and female mice were included, and analyses were designed to assess independent and interactive effects of gonadal sex, sex chromosomes, and gonadal status on fibrotic outcomes.

### Animals

Male FCG mice on a C57BL/6J background (Jackson Laboratory strain 010905) were obtained from Arthur P. Arnold and bred with C57BL/6J female mice obtained from Jackson Laboratory (Maine). Animals were housed under pathogen-free conditions with food and water *ad libitum* and aged to 16 months. All experiments and procedures were approved by the Institutional Animal Care and Use Committee at University of Miami (Protocol # 16–041) or Loyola University Chicago (Protocol # 2022026) facilities accredited by the American Association for the Accreditation of Laboratory Animal Care. Genotyping was performed according to Burgoyne and Arnold [12]. Briefly, a triplex PCR was performed on ear punch DNA to identify the presence of Sry transgene, Y chromosome, and autosomal PCR positive control gene myogenin.

In the FCG mouse strain used in the present experiments (Jackson strain 10905), the Y chromosome harbors a translocation of 9 X chromosome genes [58]. This means that any differences between XX and XY mice with the same gonads can be attributed to either sex chromosome complement (XX vs. XY, typical of wild type males and females), or to higher expression of one or more of the 9 translocated genes in XY vs. XX mice. Further work is needed to discriminate between these possibilities [59]. A new strain of FCG mice eliminates this confound (Jackson Laboratory strain 039108).

### Sex steroid hormone measurements

Serum E_2_ and testosterone concentrations were measured in each group at University of Virginia Center for Research in Reproduction Ligand Assay and Analysis Core Laboratory (Charlottesville, Va) using the estradiol Mouse and testosterone ALPCO ELISA kits.

### BLM-induced lung injury

After induction of anesthesia with ketamine, bleomycin sulfate (Sigma-Aldrich Corp; St. Louis, MO) dissolved in 50 µl sterile saline was administered by direct intratracheal instillation (2.0 units/kg). Control mice received 50 µl of intratracheal sterile saline. Mice were weighed at baseline, on day seven post-BLM, and at sacrifice. Mice were sacrificed 21 days following BLM or saline administration. Lung sections were stained with Masson’s trichrome, and pulmonary fibrosis was measured by semiquantitative Ashcroft scale.

In some experiments, FCG mice (16 months) were gonadectomized (GDX) followed by intra-tracheal BLM administration (2.0 units/kg) two weeks later. Lung tissue was obtained at 21 days post-BLM treatment.

### Histological analysis and ashcroft scoring

Right lung lobes were inflated with 10% neutral buffered formalin (NBF) under 25 cm H_2_O constant pressure. Lung tissue and 3*D ex vivo* explants were embedded in paraffin and 4 μm sections were taken for hematoxylin-eosin and Masson’s Trichrome staining. Pulmonary fibrosis was assessed by a pathologist [60] blinded to the experimental groups using the numerical Ashcroft scale [61] on Masson’s Trichrome-stained slides at 20x magnification. Individual fields were assessed by systematically moving over a 32-square grid; each field was assessed for severity of fibrosis and assigned a score of 0 (normal lung) to 8 (total fibrosis of the field). Mean ± SEM values are reported.

### Collagen content as measured by hydroxyproline assay

Left lung lobes were harvested for tissue analyses. Lung hydroxyproline assay was performed according to the manufacturer’s instructions (Hydroxyproline Assay Kit; Sigma-Aldrich, St. Louis, MO). Briefly, 2 mg lung fragments were weighed and homogenized in 100 µl of distilled water. An equal volume of 10 M HCl was added to the samples before drying at 49 °C for three hours. 50 µl of sample was loaded onto the plate and incubated overnight at 37° C. A hydroxyproline standard curve was prepared according to a standard solution (between 0 and 1 µg/well). Absorbance was measured at 557 nm, using the SoftMax Pro Software (Molecular Devices Corp; Sunnyvale, CA). Lung collagen content per mg of tissue was calculated from hydroxyproline measurement by dividing by a factor of 13.5%, as previously described [62].

### Western analysis

Lung tissue was homogenized in T-PER (Thermo Fisher Scientific; Waltham, MA) and lysates were collected. 25 µg of protein lysate were loaded onto 8% or 10% polyacrylamide gels (Thermo Fisher) and then transferred onto nitrocellulose membranes using a Trans-Blot Turbo Transfer System (Bio-Rad; Hercules, CA). Blots were blocked with 7% non-fat milk in TBS-T (0.1% Tween-20) for an hour, and then primary antibodies were diluted in 5% blocking solution and incubated overnight at 4 °C. Rabbit anti-ERα (1:1000) (Cell Signaling Technology; Danvers, MA) and rabbit anti-ERβ (1:3000) (Proteintech; Rosemont, IL) were used to detect protein. β-actin expression was determined using mouse anti-β-actin (1:5000) (Sigma-Aldrich; St. Louis, MO). Blots were washed in TBS-T and then goat anti-Mouse and anti-Rabbit HRP-conjugated secondary antibodies were used at 1:50,000 (Thermo Fisher) in 5% blocking solution. Immunoreactive bands were determined by exposing nitrocellulose blots to a chemiluminescence HRP solution (Thermo Fisher) and imaged with the ChemiDoc Imaging System (Bio-Rad). ImageJ (National Institutes of Health, Bethesda, MD) was used to determine relative density of bands. All values were corrected for corresponding β-actin band.

### Isolation of RNA and real-time polymerase chain reaction

Total RNA was extracted from 5 mg lung tissue using the Qiagen RNeasy Micro Kit (Qiagen; Hilden, Germany). Amplification and measurement of target RNA was performed on the QuantStudio 3 real time PCR system. integrin, collagen type I α1 (Col1α1), collagen type III α1 (Col3α1), and TGF-β mRNA expression were measured. TaqMan Gene Expression Assays were used to detect transcripts (Thermo Fisher) along with qScript™ XLT One-Step RT-qPCR master mix (QuantaBio; Beverly, MA). (Catalog numbers are supplied in supplement). TaqMan expression assays to detect 18 S ribosomal RNA were used as an endogenous control to normalize for variations in the isolated RNA amount. For microRNA 29a, let-7d and, -92a analyses, cDNA was generated using TaqMan™ Advanced miRNA cDNA Synthesis Kit according to the manufacturer’s instructions. Amplification of microRNA-let-7d, -29a and, -92a was performed using TaqMan Advanced miRNA assays and TaqMan Fast Advanced Master Mix (Thermo Fisher). U6 expression was used as a control for microRNA analyses, and relative expression was calculated using the comparative C(T) method [63].

### IGF-1 ELISA

Lung tissue was rinsed in phosphate-buffered saline, homogenized in appropriate lysis buffer, and subsequent freeze-thaw cycles were performed to break the cell membrane. After centrifugation, supernatant was removed and assayed according to manufacturer’s directions for IGF-1 (R&D Systems). Serum IGF-1 concentrations were measured using the same ELISA according to the manufacturer’s instructions.

### Zymography

Matrix metalloproteinase-2 (MMP-2) and MMP-9 activity were measured in lung tissue as previously described [18]. Briefly, samples and standards (Chemicon) were loaded onto 10% zymogram gels (Invitrogen-Life Technologies). Following electrophoresis, gels were incubated for 24 h at 37° C in a gelatinase solution to allow for determination of MMP-2 or-9 proteolytic activity without interference from associated tissue inhibitors. Relative MMP activity was measured by densitometry using ImageJ (National Institutes of Health, Bethesda, MD).

### Statistics

Data were analyzed by two-way ANOVA with factors of gonadal sex (ovaries vs. testes) denoted as S and sex chromosome complement (XX vs. XY) denoted as C. In gonadectomy experiments, we used a three-way ANOVA with the third factor of gonadal status (intact vs. gonadectomized, GDX). We also performed Tukey’s multiple comparison post-test. Results were considered statistically significant at *P* < 0.05.

## Data Availability

The datasets generated and/or analyzed during the current study are available from the corresponding authors on reasonable request.
